# A Rat Model Investigation of Enhanced Facial Rejuvenation via PCL Microsphere-Induced Superior Collagen Neogenesis in the Supraperiosteal Plane

**DOI:** 10.1007/s00266-025-05503-6

**Published:** 2026-02-09

**Authors:** Pai-Nien Chu, Shengjun Zhu, Xia Dai, Shihui Zhu

**Affiliations:** 1 Shanghai Juanya Aesthetic Clinic, Shanghai, 200000 China; 2 Wuhan Yixing Medical Cosmetology Clinic, Wuhan, 430000 China; 3 Chongqing Huamei Plastic Surgery Hospital, Chongqing, 400000 China; 4 Department of Burns and Plastic Surgery, Shanghai Children’s Medical Center, School of Medicine, Shanghai Jiao Tong University, 1678 Dongfang Road, Pudong New Area, Shanghai, 200127 China

**Keywords:** Cosmetic surgery, Supraperiosteal filling, Facial rejuvenation, Collagen stimulator

## Abstract

**Background:**

Age-related collagen loss and bone resorption leads to structural changes in the bone platform, affecting facial support. Fillers restore volume and structure, but their precise biological mechanisms remain unclear. This study aims to investigate the interaction between fillers and periosteum, and systematically compare the differential effects of supraperiosteal implantation of polycaprolactone (PCL) microspheres versus hyaluronic acid (HA) on collagen formation, elastin fiber production, as well as the recruitment of vascular and periosteal stem cells (PSCs).

**Methods:**

Supraperiosteal implants of PCL microspheres and HA were evaluated in a rat model. Histological analyses, including Hematoxylin and Eosin (H&E) and Masson’s trichrome staining, were used to assess collagen ingrowth. Immunofluorescence staining was employed to identify the presence of type I and type III collagen. Elastin van Gieson (EVG) staining was used to evaluate elastin fiber formation. Additionally, the recruitment of Emcn-positive blood vessels and LepR-positive PSCs was assessed.

**Results:**

H&E and Masson’s trichrome staining revealed significantly enhanced collagen ingrowth in PCL microspheres compared to HA. Immunofluorescence staining showed substantial infiltration of type I and type III collagen within the PCL microspheres, with a predominance of type I collagen. EVG staining indicated that PCL microspheres significantly promoted elastin fiber formation. Furthermore, PCL microsphere implants facilitated notable ingrowth of Emcn-positive blood vessels and LepR-positive PSCs.

**Conclusions:**

Compared to HA, PCL microspheres more effectively stimulate collagen and elastin formation and recruit vascular and periosteal stem cells. These findings suggest PCL may offer superior regenerative potential for supraperiosteal aesthetic applications.

**No Level Assigned:**

This journal requires that authors assign a level of evidence to each submission to which Evidence-Based Medicine rankings are applicable. This excludes Review Articles, Book Reviews, and manuscripts that concern Basic Science, Animal Studies, Cadaver Studies, and Experimental Studies. For a full description of these Evidence-Based Medicine ratings, please refer to the Table of Contents or the online Instructions to Authors www.springer.com/00266.

## Introduction

Facial augmentation is a widely used technique in aesthetic medicine, aimed at addressing age-related changes, such as soft tissue sagging, volume loss, and the consequent hollowed appearance [1]. This procedure involves injecting fillers into various facial layers, including the subcutaneous dermal layer, the fat layer, and the periosteal layer. Although numerous studies have demonstrated successful outcomes from augmenting the dermis and subcutaneous fat layers, research on upper periosteal layer augmentation remains limited. Correction of the supraperiosteal region is also significant, as it is closely influenced by alterations in underlying bony structures. Specifically, bone resorption and reduced bone density lead to diminished structural support [2]. Moreover, age-related changes in the periosteum itself further exacerbate volume depletion and contour irregularities. These factors underscore the need for more comprehensive investigations into periosteal-level augmentation to better understand its role and effectiveness in facial rejuvenation. By strategically injecting fillers along the bone structure, practitioners can optimize facial architecture. For instance, augmenting the zygomatic arch can enhance cheek prominence and improve midfacial support [3–5]. While traditional fillers like hyaluronic acid (HA) provide immediate enhancements in facial contour and firmness [6], their transient nature due to material degradation inevitably results in the reappearance of deficiencies over time, compromising long-term integrity of facial structures [7]. Therefore, it is essential to develop a long-term, effective regenerative strategy capable of actively engaging autologous tissues in restoring these structural alterations.

Collagen, a fundamental protein in the human body, confers strength and structural support to various tissues [8], including the periosteum [9]. Augmenting collagen deposition within the periosteal region is pivotal for restoring and sustaining facial contours. Elevated collagen levels not only fortify the tensile strength of the periosteum [10], thereby providing enhanced support for overlying soft tissues but also contribute to a more uplifted and youthful appearance. Apart from aesthetic enhancements, collagen augmentation amplifies the reparative capacity of aging microenvironment, presenting promising avenues for non-surgical facial rejuvenation.

Recent advancements in aesthetic medicine have highlighted the significant potential of supraperiosteal fillers in stimulating collagen neogenesis and enhancing tissue regeneration, with particular attention being drawn to polycaprolactone (PCL)-based products such as Ellansé^®^ [11–17]. Comprising 30% PCL microspheres and 70% carboxymethyl cellulose (CMC), this biodegradable and biocompatible filler exhibits immediate volumizing effects while also inducing long-term collagen stimulation. The multifunctionality of PCL microspheres positions them as a versatile and efficacious tool in non-surgical cosmetic treatments, harnessing the body's innate capacity for collagen regeneration to restore facial volume, improve skin elasticity, and create a more well-defined youthful contour. However, the regulatory mechanisms of fillers on periosteal cells remain elusive, presenting a significant challenge due to the potential risk of ectopic ossification resulting from prolonged retention within the periosteum.

The periosteal region, abundant in periosteal stem cells (PSCs) [18], plays a crucial role in regeneration and repair by serving as a primary source of autologous collagen production. In this study, we systematically evaluate the respective abilities of traditional HA-based fillers and collagen-stimulator PCL-based fillers to induce collagen neogenesis post-periosteal implantation. Through comparing the recruitment of key periosteal stem cells and the induction of neovascularization, we elucidate the critical mechanisms that enhance the regenerative microenvironment. This research provides a comprehensive experimental basis for implementing aesthetic fillers within the periosteum and explores the underlying mechanisms facilitating facial skeletal augmentation to address age-associated volume loss and contour deformation.

## Materials and Methods

### Study Design

The studies analyzed were conducted between February and August 2024, with overlapping timelines. All experimental procedures adhered to ethical standards for animal care. Animals were lawfully acquired and their use complied with federal, state, and local regulations, as well as guidelines from the Institutional Animal Care and Use Committee (IACUC) of Shanghai SLAC Laboratory Animal Co., Ltd. Proper care, including appropriate housing, feeding, and sanitary conditions, was ensured for all animals. The study received approval from the aforementioned IACUC.

A total of 20 male Sprague-Dawley (SD) rats, each 6 weeks old, were employed in the experiments. To minimize the impact of individual animal variability on filler outcomes, either PCL-based or HA-based filler was randomly administered bilaterally along the sagittal suture. Animals were individually housed in separate cages under controlled room temperature with a 12-hour light/dark cycle. They were provided with ample water and standard feed. Random assignment was applied to all experimental subjects to ensure unbiased results.

### Materials

The PCL-based filler was obtained from AQTIS Medical B.V (Utrecht, Netherlands). The HA-based filler (Juvederm Voluma with lidocaine) was purchased from Allergan (Irvine, CA, USA). Hematoxylin and eosin (H&E) staining kit (586X and 51275) and Masson's trichrome staining kit (HT15-1KT) were acquired from Sigma-Aldrich (St. Louis, MO, USA). The collagen fiber and elastic fiber staining kit (EVG-Weigert) were procured from Solarbio Life Sciences (Beijing, China). The primary antibodies of rabbit anti-rat type III collagen (ab184993) and rabbit anti-rat osteopontin (OPN, ab8448) were purchased from Abcam (Cambridge, UK). Rabbit anti-rat type I collagen primary antibody (NB600-408) was obtained from Novus Biologicals (Littleton, CO, USA). Mouse Leptin Receptor (LepR) biotinylated antibody (BAF497) was sourced from R&D systems (Minneapolis, MN, USA). Rat anti-rat endomucin (Emcn) was obtained from Santa Cruz Biotechnology (Piscataway, NJ, USA). All secondary antibodies were procured from Jackson ImmunoResearch Laboratories (West Grove, PA, USA).

### Construction of a Rat Model of Supraperiosteal Implantation

The six-week-old male Sprague-Dawley rats were anesthetized using 3.5% isoflurane at a flow rate of 0.5 L/min, and the fur on their heads was carefully removed to facilitate the surgical procedure. After disinfection with iodophor, a 2 cm longitudinal incision was made along the midline suture using anatomical scissors to ensure precise delivery of the filler to the periosteum rather than the dermis. Careful dissection was performed to separate the underlying diaphragm, ensuring minimal trauma to surrounding tissues. Subsequently, a 27 G syringe needle was carefully inserted into the supraperiosteal plane. A total volume of 0.5 mL of either PCL-based or HA-based filler was randomly administered bilaterally along the sagittal slit. The multi-point injection technique was employed to ensure even distribution and optimal efficacy. Specifically, the total volume was divided into five distinct injection sites, with each site receiving exactly 0.1 mL of the filler. Finally, the incision site was meticulously closed and sutured to ensure proper healing and minimize post-operative complications.

### Histological Analysis

After a feeding period of 4 and 16 weeks, the experimental rats were euthanized by neck dislocation. The intact skull was meticulously dissected, washed with PBS, immersed in 4% paraformaldehyde for fixation over a duration of 72 h. Following an hour-long PBS rinse, decalcification was performed using 0.5 M neutral ethylene diamine tetraacetic acid (EDTA) for a total of 8 weeks with weekly replacement of fresh decalcification solution. Once complete decalcification was achieved, the skull tissue underwent careful trimming and stepwise dehydration using gradient alcohol before being substituted with xylene and subsequently embedded in molten paraffin for three hours to prepare tissue wax blocks. Sections measuring at a thickness of 4.5 μm were obtained from these samples utilizing the Leica tissue microtome. Subsequently, H&E staining, Masson's trichrome staining, and EVG staining were conducted according to the manufacturer’s instructions, respectively. Finally, the stained sections were captured and documented using the Olympus slide scanning system.

### Immunofluorescence Analysis

For analysis of the changes in the local microenvironment following implantation, we utilized immunofluorescence staining to assess collagen formation, focusing on the recruitment of PSCs and angiogenesis surrounding the fillers. The procedure began with fixed and decalcified skull tissues, which were first rinsed in PBS and then immersed overnight in a 20% sucrose solution containing 2% polyvinylpyrrolidone to ensure adequate tissue preservation. Subsequently, the tissues were embedded in OCT compound and sectioned into 10 μm slices using a Leica cryostat.

For the immunofluorescence staining process, the sections were permeabilized with 0.1% Triton X-100 and then blocked with 5% goat serum at room temperature for 1 hour to prevent nonspecific binding. The sections were incubated overnight at 4 °C with primary antibodies targeting type I collagen, type III collagen, Emcn, OPN, and LepR. Following primary antibody incubation, the sections were washed three times with PBST (PBS containing 0.1% Tween 20) to remove any unbound antibodies. The sections were then incubated with secondary antibodies for 1 hour at room temperature to enable visualization. Finally, the samples were mounted using ProLong Gold Antifade Reagent with DAPI (Cell Signaling Technology) to preserve fluorescence and counterstain the nuclei. High-resolution imaging was performed using a Leica confocal microscope to capture detailed images of the stained tissue sections, providing insights into the microenvironmental changes post-implantation. The relative fluorescence intensity of the specific protein was calculated using ImageJ software.

### Statistical Analyses

Statistical analyses were conducted using GraphPad PRISM software (version 9.0; GraphPad, La Jolla, CA, USA). Data are presented as mean ± standard deviation (SD). The number of biological replicates (N) reflects experiments conducted at least three times, unless specified otherwise. For comparisons between two groups, unpaired two-tailed Student’s *t*-tests were employed when appropriate. A *P*-value of less than 0.05 was considered statistically significant, indicated as follows: *P < 0.05; **P < 0.01; ***P < 0.005; ****P < 0.001.

## Results

### PCL-Based Fillers Effectively Facilitated the Long-Term Promotion of Collagen Fiber Formation in the Periosteum

**Fig. 1:**
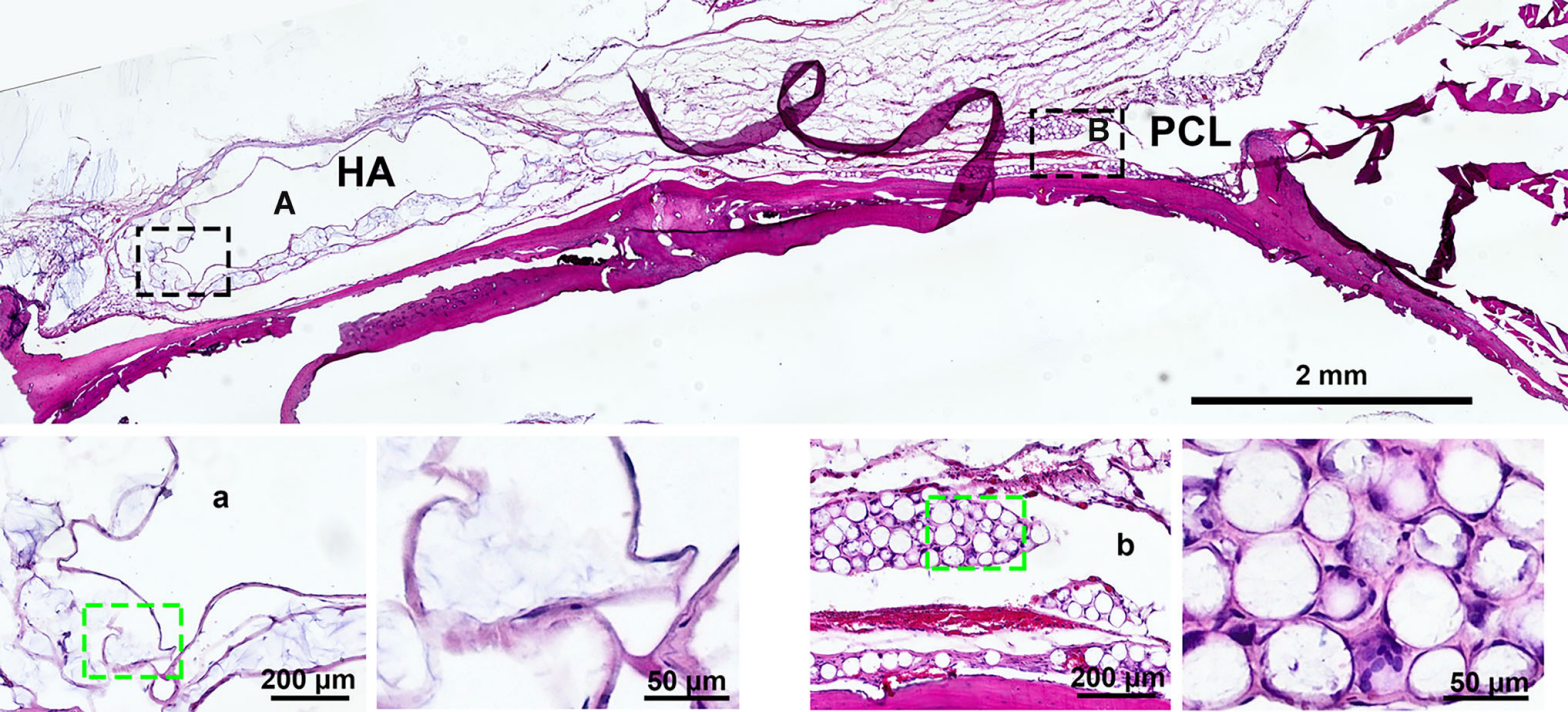
Representative H&E staining of HA or PCL filler following implantation in the periosteum of rat skull for a duration of 4 months. **A** HA filler; **B** PCL filler. The bottom image depicts an enlarged view of the area enclosed by the dotted box. The scale bar in the large-field image above represents 2 mm, while the magnified inset in the figure below corresponds to 200 μm and 50 μm, respectively

**Fig. 2:**
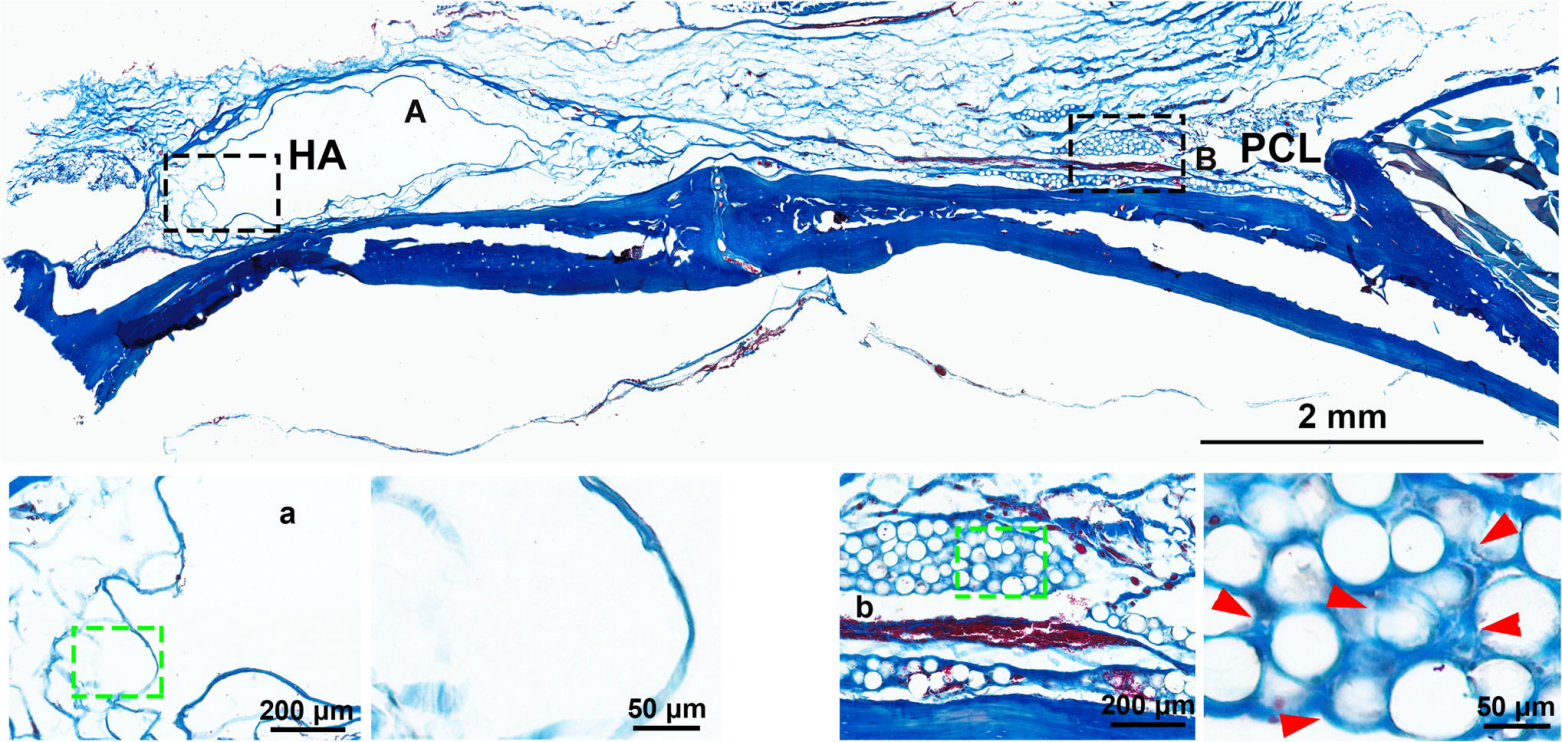
Representative Masson’s trichrome staining of HA or PCL filler following implantation in the periosteum of rat skull for a duration of 4 months. **A** HA filler; **B** PCL filler. The bottom image depicts an enlarged view of the area enclosed by the dotted box. Red arrow, collagen fibre. The scale bar in the large-field image above represents 2 mm, while the magnified inset in the figure below corresponds to 200 μm and 50 μm, respectively.

In a comprehensive 4-month study, H&E staining showed significantly greater cellular infiltration around PCL microspheres, with robust cell proliferation and integration into surrounding tissue (Fig. 1). In contrast, HA implants were encapsulated by a dense fibrotic layer, with minimal cellular activity. This suggests that the tightly crosslinked structure of HA impedes tissue ingrowth, even in the regenerative-rich periosteal environment. Masson’s trichrome staining further confirmed these findings: while the HA group exhibited minimal blue collagen staining, the PCL group showed abundant mature collagen fibers surrounding the microspheres (Fig. 2), indicating enhanced stimulation of collagen fiber formation.

**Fig. 3:**
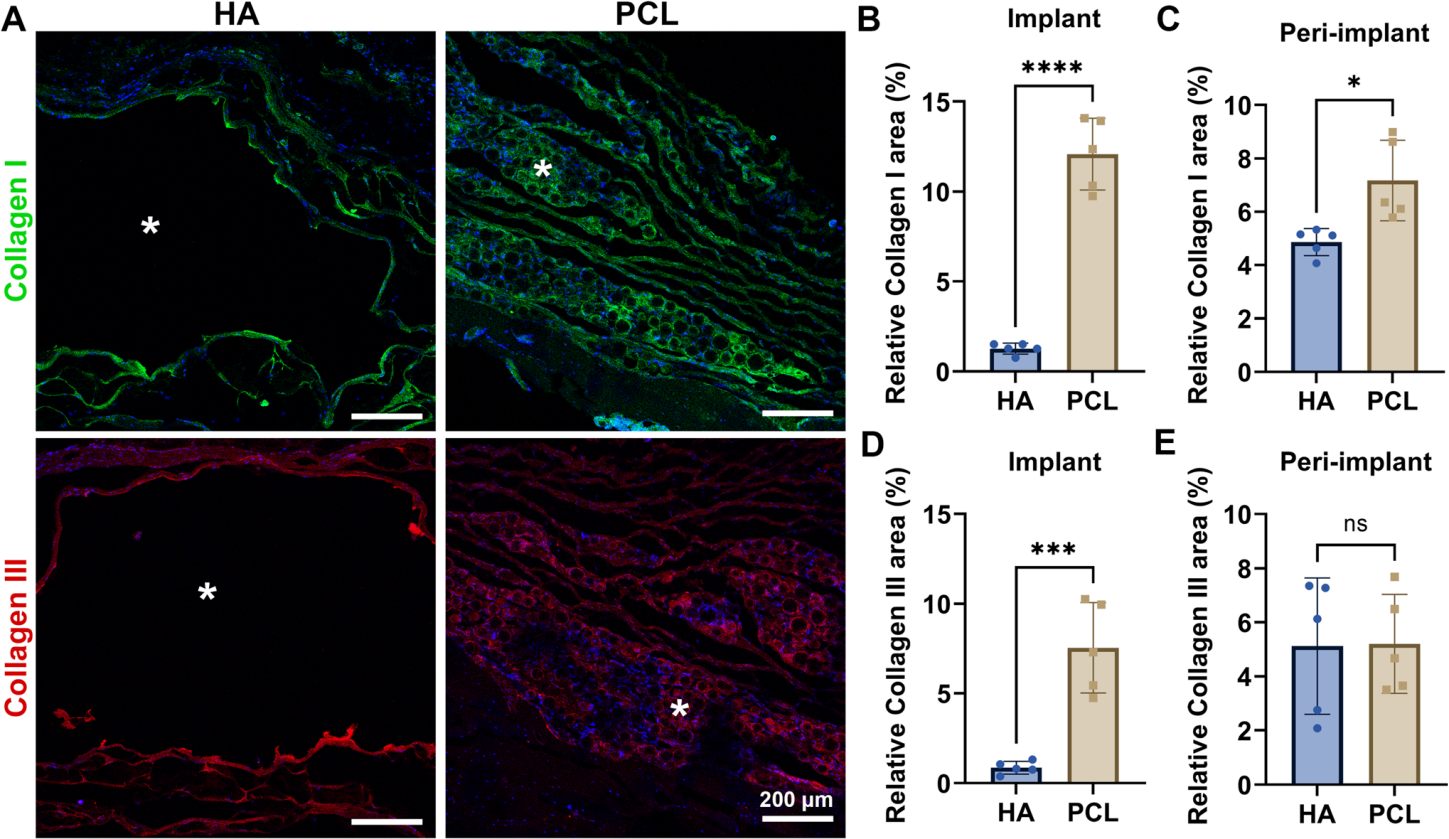
Effect of implanted fillers on collagen formation following implantation in the periosteum of rat skull for a duration of 4 months. **A** Representative immunostainings of collagen I and collagen III in HA and PCL fillers. Asterisk, implanted region. Scale bars, 100 μm. **B**, **C** Quantification of the relative collagen I area in implants (**B**) and peri-implants (**C**). **D**, **E** Quantification of the relative collagen I area in implants (**D**) and peri-implants (**E**). Data are means ± SD. *P < 0.05, ***P < 0.005, and ****P < 0.001; ns, not significant (unpaired two-tailed Student’s t test)

**Fig. 4:**
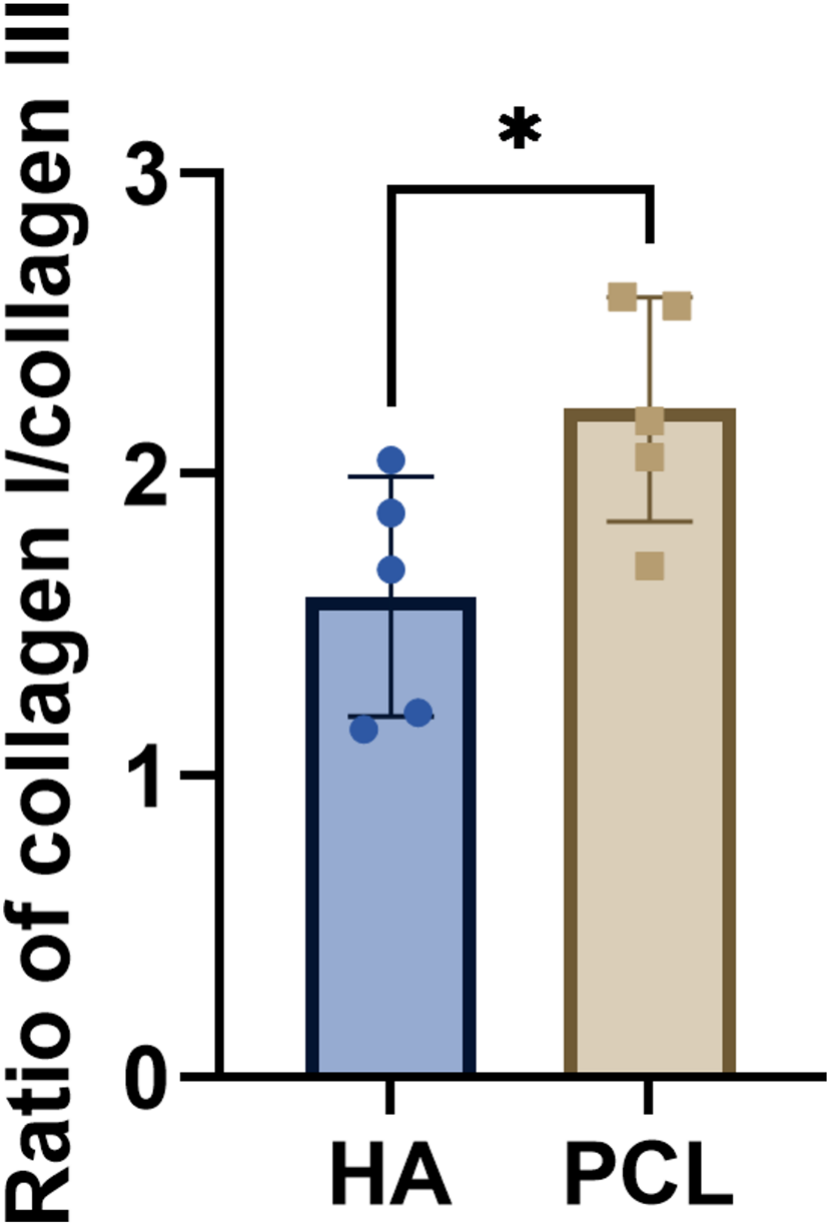
The effect of implanted fillers on the alteration of collagen formation in the periosteum of rat skull. Data are means ± SD. *P < 0.05 (unpaired two-tailed Student’s t test)

Given the key roles of type I and type III collagen in tissue remodeling—type I providing tensile strength and type III contributing to elasticity [19]—we performed immunofluorescence staining to evaluate collagen induction. PCL fillers significantly upregulated both type I and type III collagen compared to HA, particularly within the filler core (Fig. 3). However, in the peri-implant area, no significant difference in type III collagen was observed between the groups. Quantitative analysis showed that PCL fillers induced a significantly higher ratio of type I to type III collagen than HA (Fig. 4).

### PCL-Based Fillers Effectively Enhanced the Recruitment of PSCs for their Involvement in Collagen Formation

**Fig. 5:**
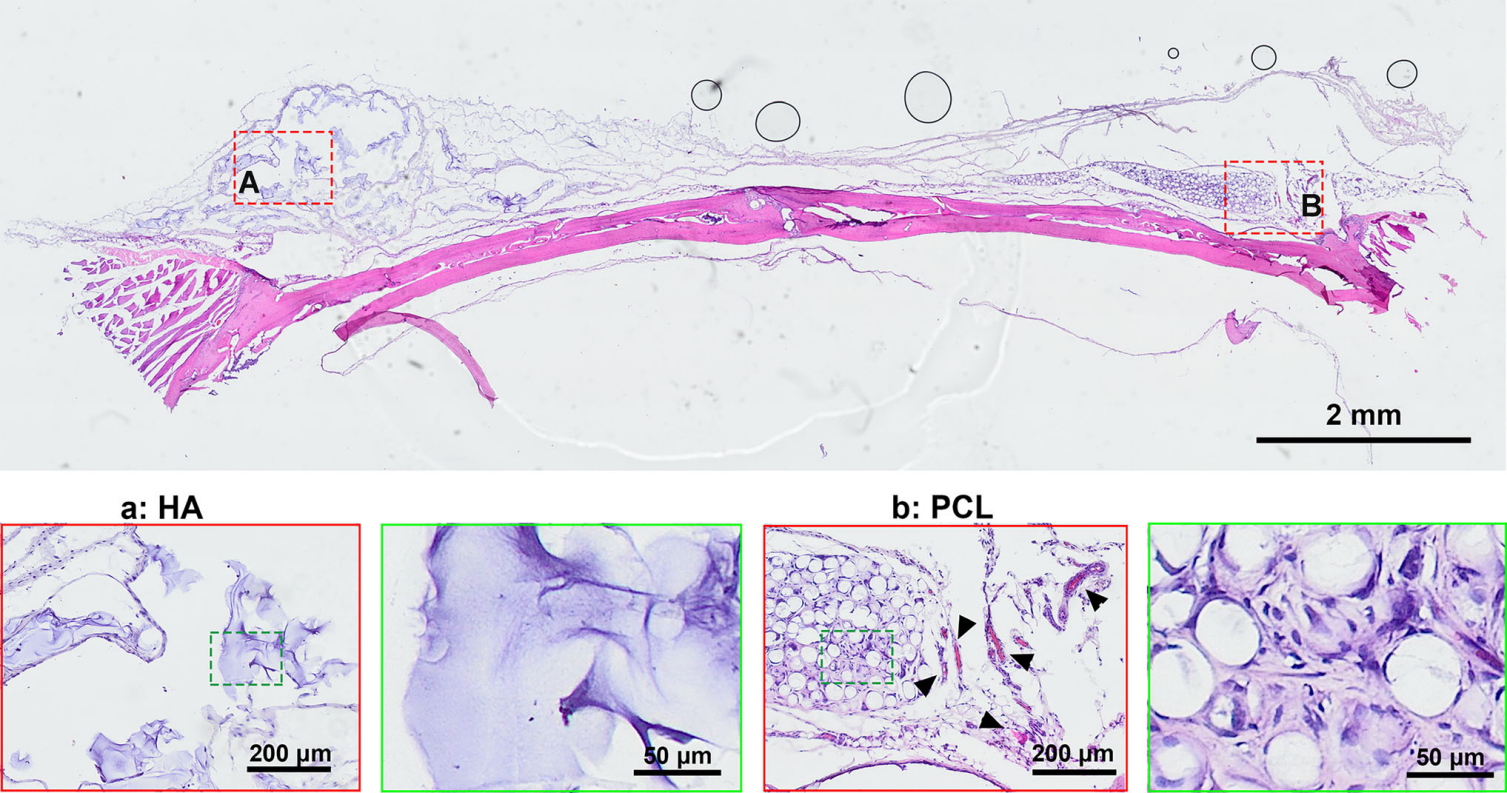
Representative H&E staining of HA or PCL filler following implantation in the periosteum of rat skull for a duration of 1 month. **A** HA-based filler; **B** PCL-based filler. Black arrow, blood vessels. The bottom image depicts an enlarged view of the area enclosed by the dotted box. The scale bar in the large-field image above represents 2 mm, while the magnified inset in the figure below corresponds to 200 μm and 50 μm, respectively.

**Fig. 6:**
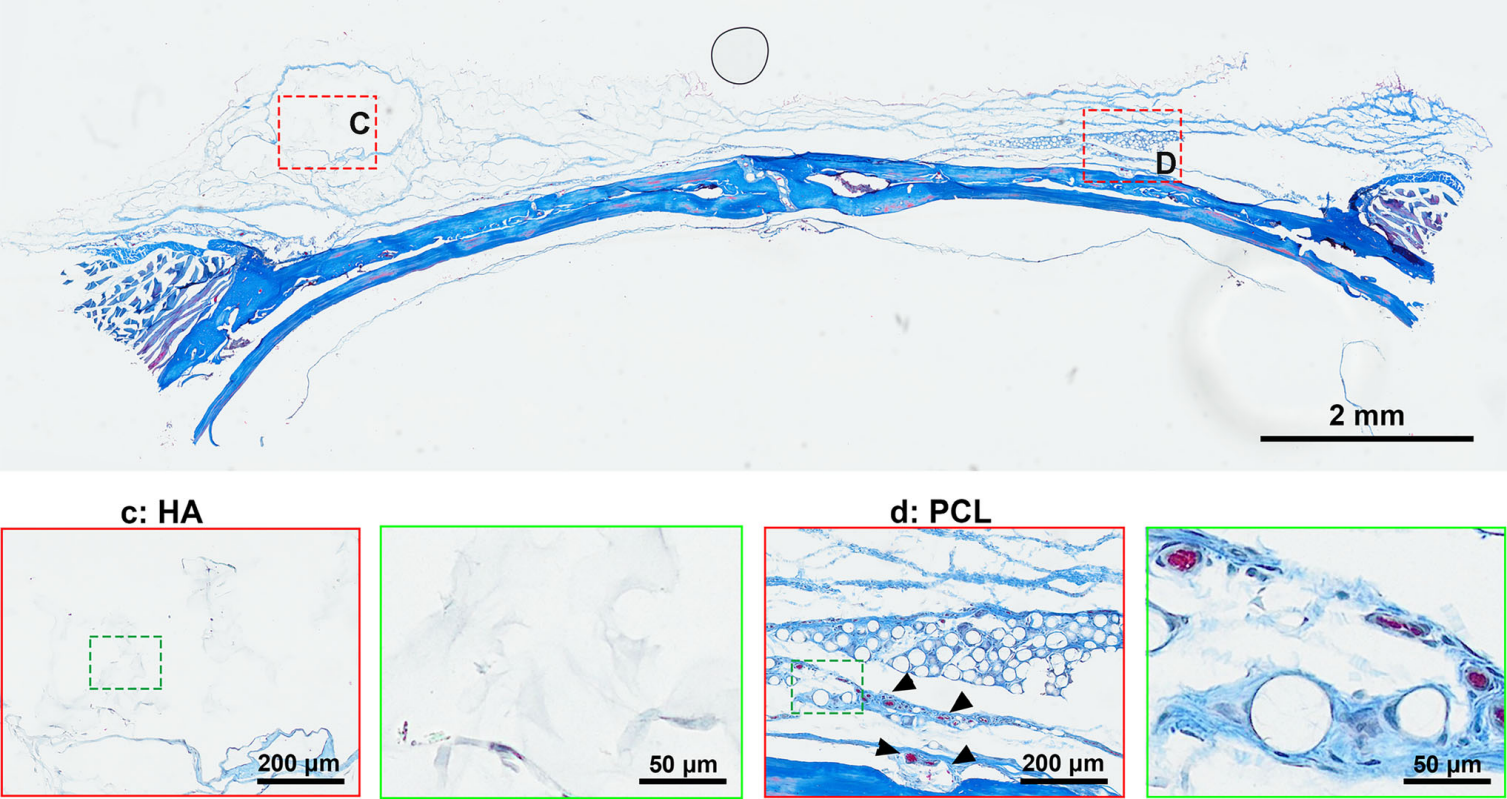
Representative Masson’s trichrome staining of HA or PCL filler following implantation in the periosteum of rat skull for a duration of 1 month. **C** HA-based filler; **D** PCL-based filler. The bottom image depicts an enlarged view of the area enclosed by the dotted box. The scale bar in the large-field image above represents 2 mm, while the magnified inset in the figure below corresponds to 200 μm and 50 μm, respectively

In the comprehensive 1-month study, H&E staining revealed that HA fillers were surrounded by a dense scar layer with minimal cellular infiltration and limited degradation (Fig. 5). In contrast, PCL fillers exhibited substantial tissue ingrowth, with fibroblastic cells surrounding the microspheres. Notably, extensive neovascularization was observed in the PCL group, supporting ongoing cellular proliferation and metabolic activity (Fig. 5). Masson’s trichrome staining further showed abundant early collagen deposition and increased vascularization around PCL microspheres, while the HA group showed negligible collagen formation (Fig. 6). These findings indicate that PCL fillers effectively stimulate early periosteal collagen production, unlike HA fillers.

**Fig. 7:**
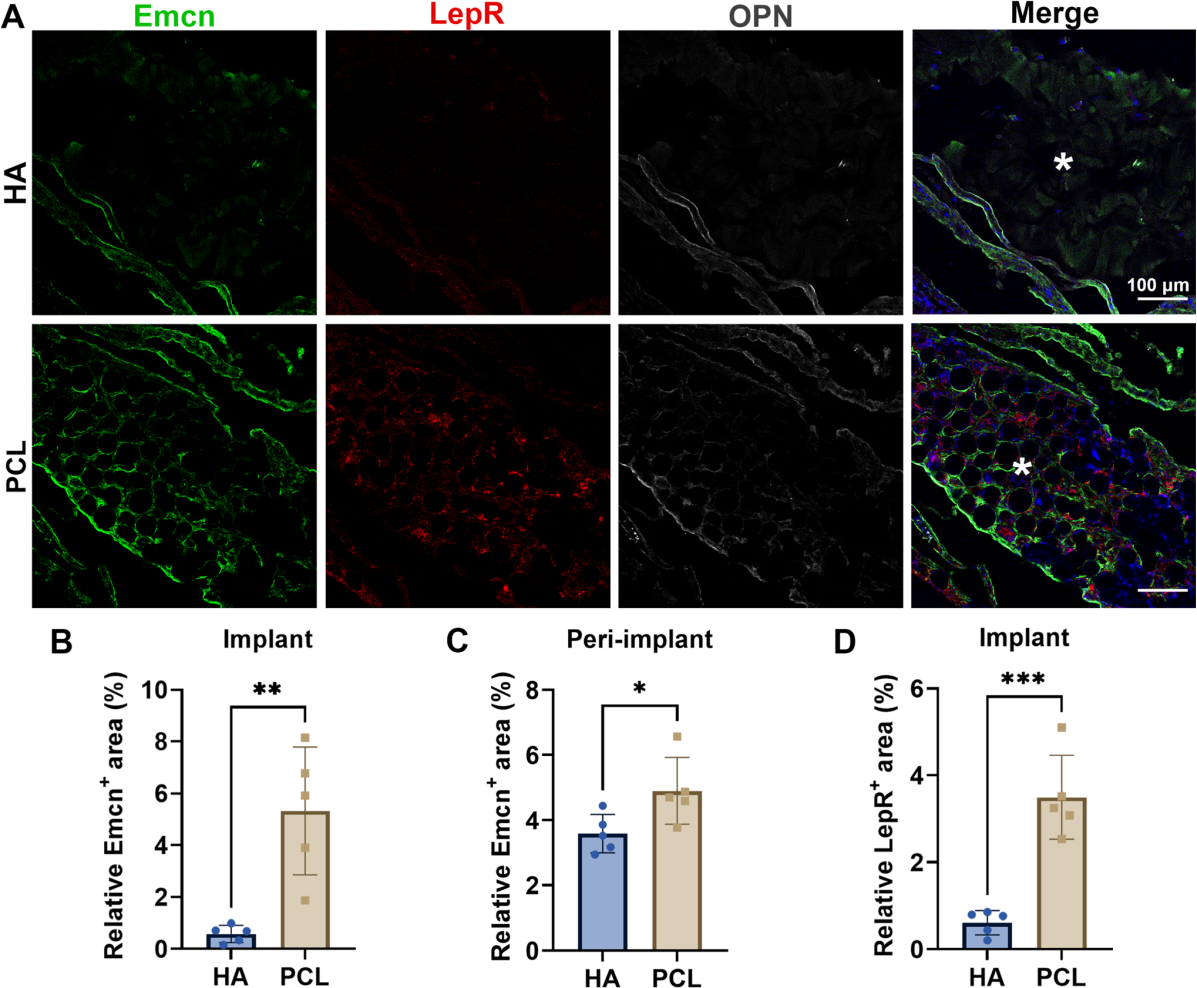
Effect of implanted fillers on the recruitment of PSCs and ingrowth of blood vessels following implantation in the periosteum of rat skull for a duration of 1 month. Scale bars, 100 μm. **A** Representative immunostainings of LepR+ PSCs, Emcn+ vessels, and OPN+ mature osteoblast in HA and PCL fillers. **B**, **C** Quantification of the relative Emcn+ vessel area in implants (**B**) and peri-implants (**C**). **D** Quantification of the relative LepR+ area in implants. Data are means ± SD. *P < 0.05, and ***P < 0.005 (unpaired two-tailed Student’s t test)

The periosteum is a highly regenerative tissue rich in blood vessels, immune cells, osteoblasts, and mesenchymal stem cells [18, 20–22]. Given the differential collagen-inducing capacity of HA and PCL fillers, we further examined their influence on the periosteal microenvironment, particularly focusing on periosteal stem cell (PSC) recruitment. Using Emcn staining to label neovasculature, we found that vascular ingrowth was limited to the periphery of HA fillers (Fig. 7A–C), while PCL fillers showed extensive Emcn-positive neovascularization throughout the implant and surrounding tissue. Additionally, LepR^+^ PSCs were abundant in the core of PCL fillers but scarce in the HA group (Fig. 7A, D), suggesting that PCL promotes both angiogenesis and stem cell recruitment, supporting endogenous collagen synthesis.

To assess the risk of ectopic ossification, we evaluated osteogenic differentiation by staining for OPN. No significant presence of OPN^+^ osteoblasts was detected in either group, including the high-LepR regions within PCL fillers (Fig. 7A), indicating that recruited PSCs primarily differentiate into fibroblasts rather than osteoblasts. Thus, PCL-based fillers enhance collagen regeneration without triggering unwanted bone formation or tissue disruption.

### The Implantation of PCL-Based Fillers Promoted the Synthesis of Elastin in the Periosteal Tissue

**Fig. 8:**
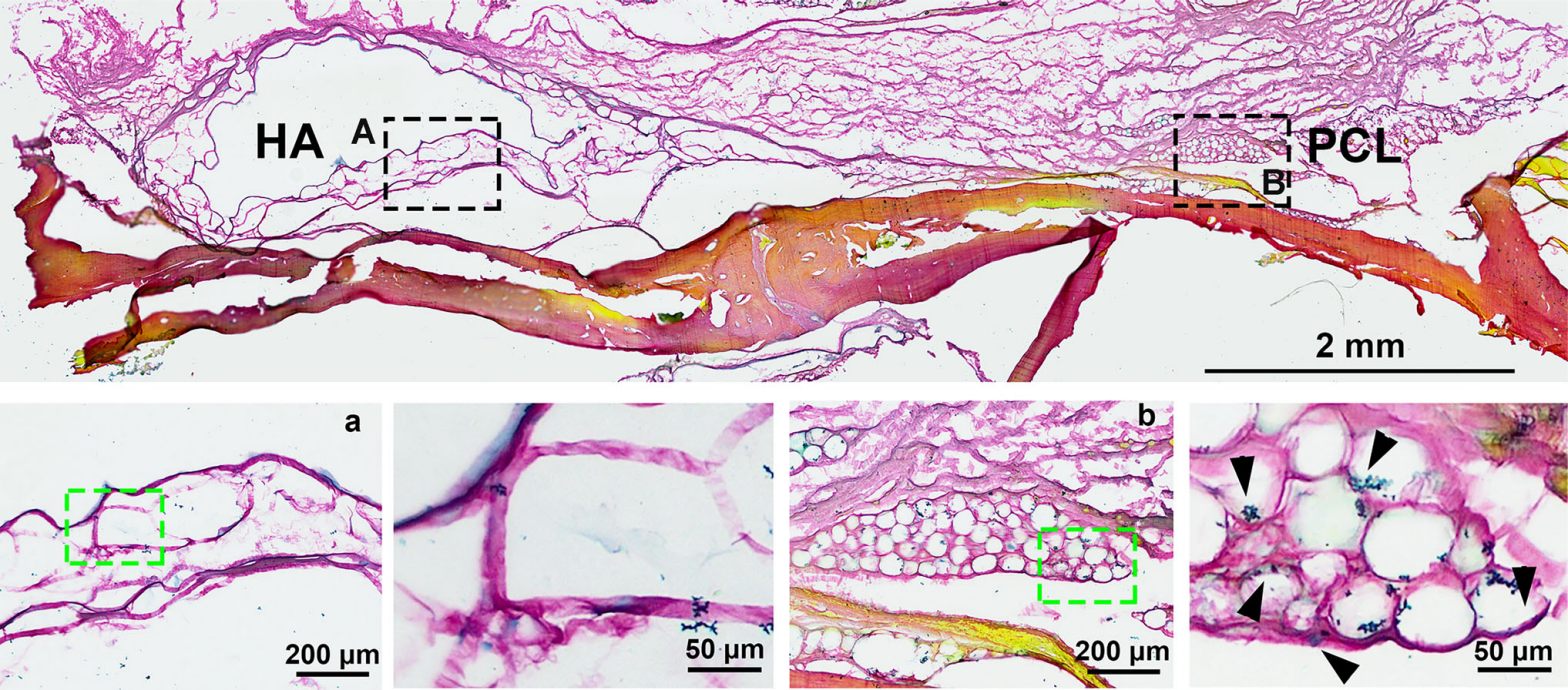
Representative EVG staining of HA or PCL filler following implantation in the periosteum of rat skull for a duration of 4 month. **A** HA filler; **B** PCL filler. The bottom image depicts an enlarged view of the area enclosed by the dotted box. Black arrow, elastin. The scale bar in the large-field image above represents 2 mm, while the magnified inset in the figure below corresponds to 200 μm and 50 μm, respectively

In aesthetic enhancement and facial rejuvenation, elastin plays a crucial role in enhancing facial contours and maintaining a firm, youthful appearance [23–25]. Therefore, we examined the effect of PCL-based fillers on elastin formation compared to HA after periosteal implantation. EVG staining showed that PCL fillers significantly promoted elastin production, whereas elastin expression was nearly absent in the HA group (Fig. 8). Notably, new elastin was found both around and within the PCL microspheres, suggesting that PCL fillers may offer superior benefits in enhancing elastogenesis, potentially leading to better facial contouring and skin tightening.

## Discussion

The supraperiosteal collagen matrix plays a crucial role in facial contouring and volume restoration by providing essential structural support and elasticity [26]. However, as individuals age, there is a significant depletion of this collagen matrix, resulting in sagging skin and reduced facial definition. Although PCL-based fillers such as Ellansé^®^ have been proven effective in stimulating autologous collagen regeneration when implanted in the dermis to address issues like skin sagging and wrinkles, limited research exists on whether supraperiosteal implantation yields similar collagen-inducing effects. In this study, we demonstrate that supraperiosteal implantation of PCL-based fillers effectively induces neocollagenesis, offering a promising approach to counteract age-related collagen loss and enhance facial rejuvenation.

The stability of fillers and the bony surface is crucial for achieving favorable outcomes in craniofacial platform filling. Following implantation, HA merely forms a fibrous layer encircling the filler, lacking strict integration with the bone interface. Consequently, there exists a risk of displacement over long-term use or improper placement, potentially leading to migration or pressure atrophy that can induce changes in the overlying skin and soft tissue. In contrast, PCL microspheres exhibit tight integration within the bone interface and are enveloped by abundant new collagen. Appropriately positioned fillers mimic the underlying bone structure, providing support for soft tissue and enhancing its aesthetic appearance. The process of neocollagenesis induced by PCL-based fillers primarily involves angiogenesis and recruitment of periosteal stem cells. Angiogenesis facilitates new blood vessel growth, supplying essential nutrients and oxygen to the implantation site while creating an optimal environment for tissue regeneration. Simultaneously, recruitment of stem cells from the periosteum plays a pivotal role in collagen synthesis as these cells differentiate into fibroblasts directly involved in producing new collagen fibers. The interplay between these biological processes underscores the efficacy of PCL-based fillers in promoting collagen renewal.

Changes in the ratio of type I to type III collagen within the periosteum also play a crucial role in regenerative repair, particularly when tissue repair or remodeling is facilitated through implanted fillers [27]. This alteration not only impacts the integration and stability of the filling material but also exhibits a close association with the final aesthetic and functional outcomes. Type III collagen confers flexibility and support to nascent tissue during its initial healing phase, constituting an indispensable component of early tissue repair processes [27]. As tissues mature and undergo remodeling, type III collagen gradually gives way to type I collagen [28], leading to a decrease in its proportion. Type I collagen assumes a critical function by providing strength and stability to tissues [29], thereby serving as an integral constituent of fully developed tissues.

Another concern pertains to the potential of supraperiosteal fillings to stimulate bone growth, given that the periosteum harbors a substantial number of cells capable of inducing osteogenesis, such as osteoblasts and periosteal stem cells [30]. Clinically, there have been reports of ectopic bone hyperplasia in some patients following periosteal grafting. However, our experimental findings revealed no discernible evidence of new bone formation in either HA or PCL implants. We conclude that the occurrence of ectopic bone hyperplasia in certain clinical cases may be attributable to cranial bone trauma during the injection procedure, whereas precise supraperiosteal placement does not provoke significant ectopic ossification. In fact, previous studies have demonstrated the challenges associated with activating the host microenvironment to secrete osteogenic factors for facilitating mesenchymal stem cell differentiation and promoting new bone generation without compromising cranial integrity [31]. Furthermore, uncontrolled ectopic ossification may give rise to facial dysmorphology [32] often accompanied by sensory nerve proliferation leading to facial pain [33].

According to the findings of this investigation, facial biostimulation treatments for promoting collagen synthesis present a viable approach to augmenting the surface area of the craniofacial platform.

## Limitation

While our study highlights the potential of PCL-based fillers for collagen regeneration and facial rejuvenation, several limitations remain. First, the rat model may not fully reflect human facial anatomy, and periosteal regeneration in rats proceeds at a faster rate compared to humans, limiting the direct extrapolation of findings. This underscores the importance of clinical trials to validate safety and efficacy. Second, future research should further investigate the relationship between the extent of collagen induction and the efficacy of dermal fillers. Quantifying this relationship could provide valuable guidance for optimizing injection dosages in clinical practice. Additionally, irreversible nodules resulting from excessive stimulation warrant careful consideration and further investigation. Finally, our emphasis on short-term outcomes underscores the need for long-term studies and the inclusion of additional collagen stimulators to enable more comprehensive comparisons.

## Conclusions

In conclusion, our study underscores the potential of PCL-based fillers to address age-related facial volume loss and contour changes by stimulating Type I collagen production. Through enhanced angiogenesis and periosteal stem cell recruitment, PCL fillers create a favorable microenvironment for collagen regeneration and facial structural restoration. Despite limitations such as the use of animal models and short-term observations, these findings lay the groundwork for future clinical research. With continued investigation, PCL fillers may become a valuable tool for long-lasting improvements in facial contour and aesthetics.
